# Novel Src/Abl tyrosine kinase inhibitor bosutinib suppresses neuroblastoma growth via inhibiting Src/Abl signaling

**DOI:** 10.18632/oncotarget.13643

**Published:** 2016-11-26

**Authors:** Shayahati Bieerkehazhi, Zhenghu Chen, Yanling Zhao, Yang Yu, Huiyuan Zhang, Sanjeev A. Vasudevan, Sarah E. Woodfield, Ling Tao, Joanna S. Yi, Jodi A. Muscal, Jonathan C. Pang, Shan Guan, Hong Zhang, Jed G. Nuchtern, Hui Li, Huiwu Li, Jianhua Yang

**Affiliations:** ^1^ Department of Labour Hygiene and Sanitary Science, College of Public Health, Xinjiang Medical University, Urumqi, Xinjiang 830011, P.R. China; ^2^ Department of Pathology, University of Texas MD Anderson Cancer Center, Houston, Texas 77030, USA; ^3^ Department of Ophthalmology, Shanghai Tenth People's Hospital, Tongji University School of Medicine, Shanghai 200072, P. R. China; ^4^ Texas Children's Cancer Center, Department of Pediatrics, Dan L. Duncan Cancer Center, Baylor College of Medicine, Houston, Texas 77030, USA; ^5^ Division of Pediatric Surgery, Texas Children's Hospital Department of Surgery, Michael E. DeBakey Department of Surgery, Dan L. Duncan Cancer Center, Baylor College of Medicine, Houston, Texas 77030, USA; ^6^ Department of Biosciences, Weiss School of Natural Sciences, Rice University, Houston, Texas 77005, USA; ^7^ Central Laboratory of Xinjiang Medical University, Urumqi, Xinjiang 830011, P.R. China; ^8^ Cancer Prevention and Research Institute, The Affiliated Tumor Hospital of Xinjiang Medical University, Urumqi, Xinjiang 830011, P.R. China

**Keywords:** neuroblastoma, bosutinib, SKI-606, Bosulif, chemotherapy

## Abstract

Neuroblastoma (NB) is the most common extracranial solid tumor in children. Aberrant activation of the non-receptor tyrosine kinases Src and c-Abl contributes to the progression of NB. Thus, targeting these kinases could be a promising strategy for NB therapy. In this paper, we report that the potent dual Src/Abl inhibitor bosutinib exerts anti-tumor effects on NB. Bosutinib inhibited NB cell proliferation in a dose-dependent manner and suppressed colony formation ability of NB cells. Mechanistically, bosutinib effectively decreased the activity of Src/Abl and PI3K/AKT/mTOR, MAPK/ERK, and JAK/STAT3 signaling pathways. In addition, bosutinib enhanced doxorubicin (Dox)- and etoposide (VP-16)-induced cytotoxicity in NB cells. Furthermore, bosutinib demonstrated anti-tumor efficacy in an orthotopic xenograft NB mouse model in a similar mechanism as of that *in vitro*. In summary, our results reveal that Src and c-Abl are potential therapeutic targets in NB and that the novel Src/Abl inhibitor bosutinib alone or in combination with other chemotherapeutic agents may be a valuable therapeutic option for NB patients.

## INTRODUCTION

Neuroblastoma (NB), the most common pediatric extracranial solid tumor [1], is responsible for over 15% of childhood cancer-related deaths [2, 3]. Over 30% of NB patients are diagnosed in infancy and approximately three-quarters of cases occur in patients under five years of age [4]. Despite the improvements in patient outcomes made over the past decades, the cure rate for patients with high-risk NB remains unacceptably low, and the overall survival rate for these patients is still inadequate at about 70% at three years [5, 6]. Consequently, additional therapeutic strategies with minimal toxicity are still needed to help patients with high-risk NB, including novel targeted agents.

Src is a member of the non-receptor tyrosine kinase family and it participates in intracellular signaling pathways and has been frequently associated with cell migration, proliferation, and apoptosis [7–9]. Multiple studies have shown that abnormally activated Src is frequently associated with the malignancy of a variety of human cancer types [10, 11]. Src functions as the upstream of several important signaling pathways, including PI3K/AKT/mTOR, MAPK/ERK, and JAK/STAT3 signaling [12–14]. Inhibition of Src-mediated downstream signaling has been reported to exhibit anti-cancer efficacy [15–17]. Overexpression of the Src kinase in advanced NB patients has been associated with poor outcomes [18], and inhibition of Src tyrosine kinase activity results in decreased cell proliferation and apoptosis induction in NB cells [19–21]. Therefore, targeting Src tyrosine kinase to cure NB is a feasible solution in NB therapy.

The proto-oncogene *ABL* (*ABL1* in humans) was first discovered from the Abelson murine leukemia virus [22] and has been identified as an oncogene that was frequently associated with the chromosome translocations in human leukemia [23]. In chronic myelogenous leukemia (CML), the translocation of *ABL* within the *BCR* (breakpoint cluster region) gene results in the generation of the fusion gene, *BCR-ABL*, which encodes a constitutively activated oncogenic tyrosine kinase Bcr-Abl [24]. The Bcr-Abl oncoprotein then contributes to aberrant cell proliferation through the activation of the Ras pathway, which allows the cells to become cancerous [25].

In contrast to the well-established role of Bcr-Abl in CML, less is known about the role of the c-Abl kinase in solid tumors [26]. In mammalian cells, the non-receptor tyrosine kinase c-Abl is encoded by *ABL1* and is tightly regulated for its role in the regulation of cell proliferation, cell survival, cell migration, etc. [27]. The c-Abl protein is located both in the nucleus and the cytoplasm and it shuttles between the nucleus and the cytoplasm continuously [28]. In the nucleus, c-Abl is activated by CDC2-mediated phosphorylation during S phase of the cell cycle and exhibits DNA-binding activity, suggesting that it may participate directly in the regulation of cell cycle control [29]. In cytoplasm, c-Abl is believed to promote cell proliferation and invasion in advanced breast cancer cells [30, 31]. In human breast cancer cells and mouse fibroblasts, c-Abl is essential for Src-induced transformation of those cells by facilitating the Src/Abl/Rac/JNK/STAT3 signaling cascade which has been considered to be important in cell transformation [32]. In addition, c-Abl is also involved in the survival pathway Src/Abl/Rac/ERK5 that is activated in human breast cancer cell lines [32]. Particularly, c-Abl inhibition by imatinib suppresses NB cell proliferation due to the increased activity and stability of the CDK inhibitor p27^KIP1^ [33], suggesting that c-Abl may play a role in the proliferation of NB cells.

Bosutinib (Bosulif, SKI-606), an orally bioavailable compound, is a second-generation tyrosine kinase inhibitor which selectively inhibits the kinase activity of Src/Abl [34, 35]. In a cell free assay, bosutinib is selective for Src over other non-Src family kinases with an IC50 of 1.2 nM, and it potently inhibits Src-dependent cell proliferation in rat fibroblasts with an IC50 of 100 nM [34]. Moreover, bosutinib blocks the phosphorylation of both c-Abl and the Bcr-Abl fusion protein, thus inhibiting their kinase activity [35]. As a dual inhibitor of Src and c-Abl, bosutinib has been approved by the United States Food and Drug Administration (FDA) for treating patients with CML [36]. However, the potential anti-tumor efficacy of the bosutinib in NB has not been tested.

In this study, we assessed the inhibitory effects of bosutinib on NB cell proliferation *in vitro* and tumor growth *in vivo*. We found that bosutinib inhibited NB cell proliferation and blocked the activation of Src and c-Abl, as well as the PI3K/AKT/mTOR, MAPK/ERK, and JAK/STAT3 signaling pathways in all NB cell lines tested. Bosutinib also suppressed the colony formation ability of those NB cell lines. Moreover, bosutinib enhanced the cytotoxic effects of doxorubicin (Dox) and etoposide (VP-16) on NB cells. *In vivo*, bosutinib inhibited NB tumor growth and induced tumor cells death by abrogating Src and c-Abl mediated signaling. Taken together, these results suggest that Src and c-Abl are potential therapeutic targets in NB and that novel Src/Abl inhibitors like bosutinib alone or in combination with other chemotherapeutic agents may be promising treatment strategies for treating NB patients.

## RESULTS

### Bosutinib shows cytotoxic effects on a panel of NB cell lines

**Figure 1:**
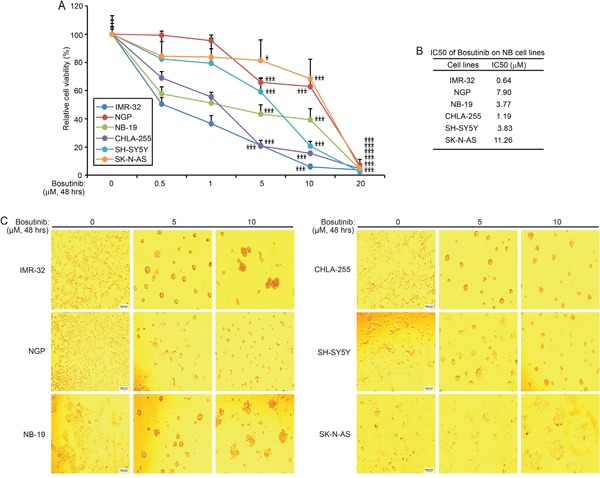
Bosutinib inhibits NB cell proliferation in a panel of NB cell lines **A.** Six NB cell lines, IMR-32, NGP, NB-19, CHLA-255, SH-SY5Y, and SK-N-AS, were treated with increasing concentrations of bosutinib for 48 hrs. Cell viability was measured by performing the Cell Counting Kit-8 (CCK-8) assay. *P*-values <0.05 (*), or *P* <0.001 (***) (Student's t-test, two-tailed) were indicated. **B.** The IC_50_ values of bosutinib on each NB cell line were calculated by using Graphpad prism 5.0. **C.** Morphologic changes of six NB cell lines treated with two concentrations of bosutinib for 48 hrs were shown, with bosutinib showing cytotoxic effects on all above cell lines.

To test whether bosutinib has cytotoxic effects on NB cell lines, six cell lines, IMR-32, NGP, NB-19, CHLA-255, SH-SY5Y, and SK-N-AS, were treated with increasing concentrations of bosutinib for 48 hrs. Cell viability of all six cell lines tested was significantly diminished by bosutinib in a dose dependent manner (Figure 1A), with IC_50_'s as shown (Figure 1B). IMR-32 cells were the most sensitive to bosutinib with an IC_50_ of 0.64 μM, while SK-N-AS cells were relatively resistant to bosutinib treatment (IC_50_ = 11.26 μM). Cell morphology changes were captured at the end of the treatment, which confirmed bosutinib-induced cytotoxicity in the tested NB cells (Figure 1C). In all cell lines tested, treatment of bosutinib at 5 μM or 10 μM for 48 hrs resulted in abnormal cell morphology. These results indicated that bosutinib suppressed the viability of NB cell lines in a dose-dependent manner.

### Bosutinib suppresses colony formation capability in NB cells

**Figure 2:**
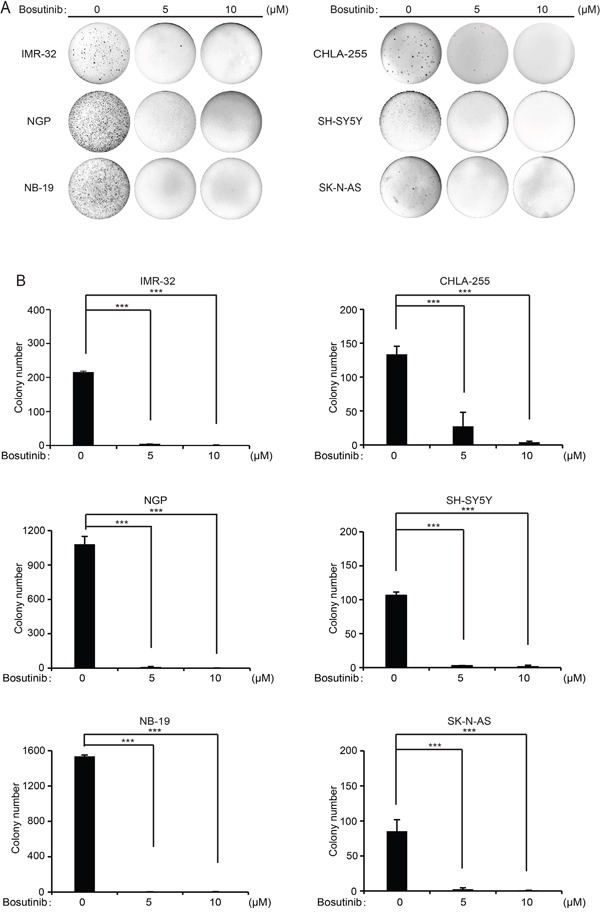
Bosutinib suppresses the colony formation ability of six NB cell lines **A.** A subset of six NB cell lines was seeded in 6-well plates in soft agar with increasing concentrations of bosutinib and allowed to grow for two to three weeks. Then, crystal violet staining was performed and the images were captured. **B.** Colony numbers from (A) were presented as mean ± S.D. *P*-values <0.001 (***) (Student's t-test, two-tailed) were indicated.

One of the distinctive features of tumor cells is that they have the ability to grow colonies in soft agar cultures. To evaluate whether bosutinib can inhibit the colony formation capability of NB cells, we performed soft agar assays of a subset of NB cell lines. Consistently, compared with the untreated control groups, the bosutinib treatment groups showed suppressed colony formation ability in all the cell lines tested (Figure 2A). Colony numbers were counted in each group, and fewer colonies were present in the bosutinib-treated groups (Figure 2B). Taken together, these results demonstrate that anchorage-independent growth of all tested NB cells was inhibited by bosutinib.

### Bosutinib inhibits Src and c-Abl activities, as well as the activation of PI3K/AKT/mTOR, MAPK/ERK, and JAK/STAT3 signaling in NB cells

**Figure 3:**
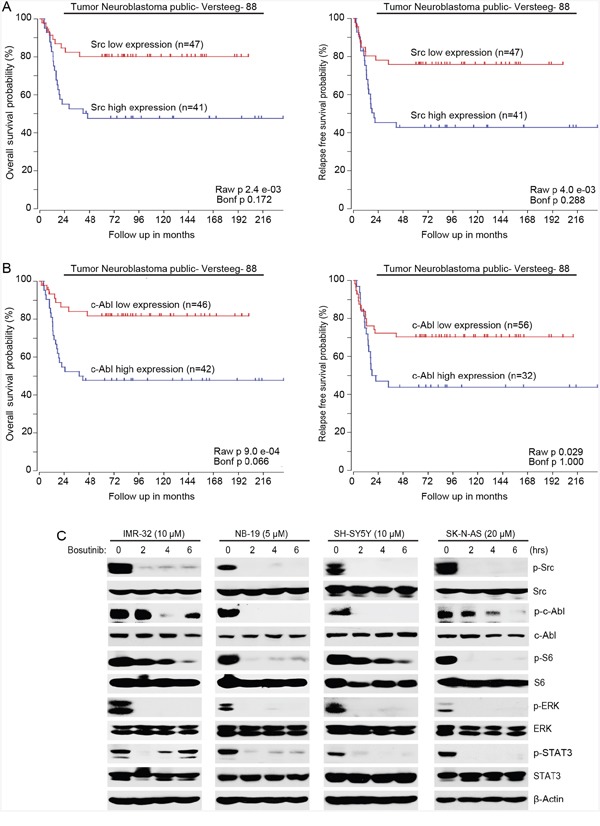
Bosutinib inhibits the phosphorylation of Src, c-Abl and the activities of the PI3K/AKT/mTOR, MAPK/ERK, and JAK/STAT3 signaling pathways in NB cells **A.** Overall survival probability for NB patients with high Src expression (blue, n=41) and low Src expression (red, n=47) (Versteeg-88 data set) were shown. Relapse-free survival probability for NB patients with high Src expression (blue, n=41) and low Src expression (red, n=47) (Versteeg-88 data set) were shown. **B.** Overall survival probability for NB patients with high c-Abl expression (blue, n=42) and low c-Abl expression (red, n=46) (Versteeg-88 data set) were shown. Relapse-free survival probability for NB patients with high c-Abl expression (blue, n=32) and low c-Abl expression (red, n=56) (Versteeg-88 data set) were also shown. High expression levels of both Src and c-Abl correlate with the poor outcome of NB patients. **C.** IMR-32, NB-19, SH-SY5Y, and SK-N-AS cells were treated with indicated concentrations of bosutinib for 0-8 hrs and the cells were lysed and subjected to immunoblotting with the indicated antibodies. β-Actin was used as a loading control in all experiments.

First, to determine whether Src/Abl had prognostic value in NB, data analysis of the R2 database (http://r2.amc.nl) was performed. The results demonstrate that high expression of both *ABL* and *SRC* genes predict lower overall and relapse-free survival in the Versteeg-88 dataset (Figure 3A-3B), which suggests that expression levels of both Src and c-Abl tyrosine kinases could be used as predictive markers for the outcomes of NB patients.

Then we investigated the molecular mechanisms that were responsible for the cytotoxicity of bosutinib in NB cells. Four NB cell lines (IMR-32, NB-19, SH-SY5Y, SK-N-AS) were treated with bosutinib for various time points (0-6 hrs) and the cells were harvested for protein immunoblotting assay. As shown in Figure 3C, bosutinib significantly decreased the phosphorylation levels of p-Src (Y416) and p-c-Abl (Y245) in all NB cell lines tested. Bosutinib also blocked the phosphorylation of p-S6 (S235/236) and p-ERK (T202/Y204) in the tested cell lines (Figure 3C). In addition, we examined levels of p-STAT3 as a readout of JAK/STAT3 signaling and found that bosutinib treatment led to a decrease in phosphorylation levels of STAT3 (Y705) in all four NB cell lines (Figure 3C). These data show that bosutinib inhibits the activities of Src, c-Abl, and the PI3K/AKT/mTOR, MAPK/ERK, and JAK/STAT3 signaling pathways in NB cells.

### Bosutinib enhances the cytotoxic effects of Dox and VP-16 on NB cells

**Figure 4:**
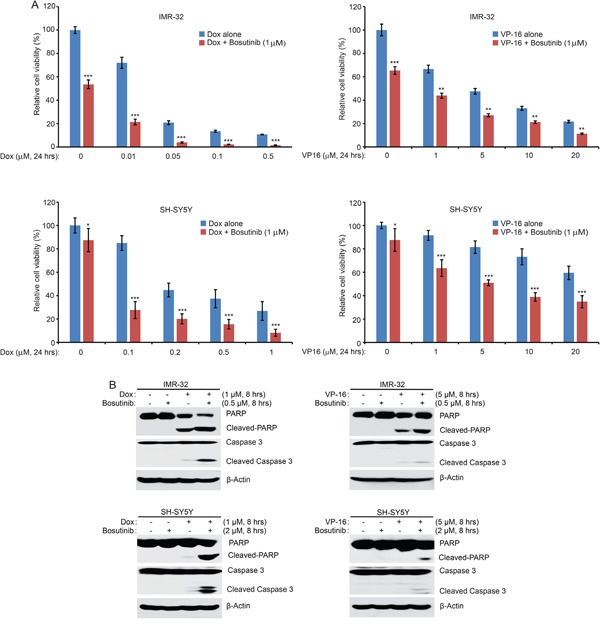
Bosutinib enhances the cytotoxic effects of Dox and VP-16 in NB cells **A.** IMR-32 and SH-SY5Y cells were seeded in 96-well plates and were incubated with the indicated concentrations of Dox or VP-16 plus DMSO or bosutinib (1 μM) for 24 hrs. Cell viability was assessed by the CCK-8 assay. Results are represented as % vehicle ± SD. *P*-values <0.05 (*), *P* <0.01 (**), or *P* <0.001 (***) (Student's t-test, two-tailed) were indicated. **B.** IMR-32 and SH-SY5Y cells were treated with either Dox (1 μM), VP-16 (5 μM), or bosutinib (0.5 μM or 2 μM) alone or in combinations for 8 hrs. All samples were then collected, subjected to SDS-PAGE, and immunoblotted with PARP and Caspase 3 antibodies. β-Actin was used as a loading control for whole cell extracts in all experiments.

As bosutinib effectively inhibited NB cell proliferation in NB cells, we reasoned that bosutinib may sensitize NB cells to the treatments of conventional agents like Dox and VP-16. To test this hypothesis, two cell lines, IMR-32 and SH-SY5Y, were treated with either Dox or VP-16 or in combination with bosutinib for 24 hrs. The results showed that the combination of bosutinib with Dox or VP-16 demonstrated greater anti-proliferative effect than either agent alone on the proliferation of both cell lines (Figure 4A). Consistently, bosutinib enhanced Dox- and VP-16-induced apoptosis by increasing Caspase 3 and PARP cleavages in the combination treatment groups of IMR-32 and SH-SY5Y cells (Figure 4B). These results indicate that bosutinib enhances Dox- and VP-16 -induced cytotoxicity in NB cells.

### Bosutinib shows anti-tumor efficacy in an orthotopic xenograft NB mouse model

**Figure 5:**
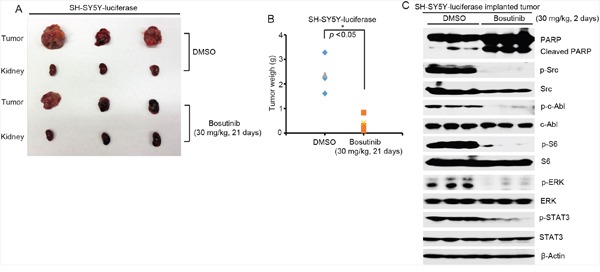
Bosutinib inhibits tumor growth in an orthotopic xenograft NB mouse model by blocking the activities of Src, c-Able and the downstream PI3K/AKT/mTOR, MAPK/ERK, and JAK/STAT3 signaling pathways **A.** At the end of treatment, images of SH-SY5Y xenografted tumors and control kidneys from DMSO control group and bosutinib treated group were taken, as shown. **B.** SH-SY5Y xenograft tumor weights from control (n=3) and treatment (n=3) groups were presented. *P*-value <0.05 (*) (Student's t-test, two-tailed) was indicated. Bosutinib significantly suppressed NB tumor growth in this mouse model. **C.** Six-week-old tumor bearing mice were treated with 30 mg/kg of bosutinib by i.p. injection once daily for two days. The mice were then sacrificed, and the tumors were harvested and lysed for immunoblotting with the indicated antibodies. β-Actin was used as a loading control.

Since bosutinib showed clear inhibitory effects on NB cell lines *in vitro*, we then tested the *in vivo* efficacy of bosutinib in a well-established NB mouse model. In this set of experiments, SH-SY5Y cells with stable luciferase expression were injected into the left kidneys of nude mice. Two weeks after injection, bioluminescent imaging was used to detect tumor signals. The tumor bearing mice were standardized by a threshold of 1 × 10^6^ total flux (p/s), and the mice, grouped by similar tumor size, were then treated with either bosutinib (30 mg/kg) or an equal volume of dimethyl sulfoxide (DMSO) daily for three weeks. At the end of treatment, mice in each group were sacrificed, and xenograft tumors were dissected and weighed. Significant tumor growth inhibition was observed in bosutinib treatment group compared to the DMSO control group (Figure 5A-5B).

To assess the signaling changes that occurred *in vivo* as a result of bosutinib treatment, six-week-old SH-SY5Y xenografted mice were treated with either bosutinib (30 mg/kg) or an equal volume of DMSO daily for two days. After the treatment, tumor tissues from both groups were harvested and lysed to perform the protein immunoblotting assay. Bosutinib significantly induced cell death in the tumor cells by inducing PARP cleavages (Figure 5C). Moreover, bosutinib significantly blocked the phosphorylation levels of p-c-Abl (Y245), p-Src (Y416), p-ERK (T202/Y204), p-S6 (Thr235/236), and p-STAT3 (Y705) in the treatment group (Figure 5C). Taken together, these results indicate that bosutinib effectively inhibits NB tumor growth *in vivo* by blocking the activities of Src and c-Abl and by blocking the PI3K/AKT/mTOR, MAPK/ERK, and JAK/STAT3 signaling pathways.

## DISCUSSION

The Src kinase has been reported to promote NB cell proliferation through Src-mediated PI3K/AKT/mTOR, MAPK/ERK, and JAK/STAT3 signaling [37, 38]. Other studies have shown that Src/Abl tyrosine kinase inhibitor dasatinib exerts anti-tumor effects in both NB cells and an orthotopic mouse model [19]. Herein we report that the novel dual Src/Abl inhibitor bosutinib inhibited NB cell proliferation in a time dependent manner and impaired anchorage-independent growth of NB cells. Bosutinib also suppressed the activities of the Src and Src-mediated PI3K/AKT/mTOR, MAPK/ERK, and JAK/STAT3 signaling pathways in NB cells.

The Abl family of protein kinases consists of two vertebrate paralogs, c-Abl (encoded by *ABL1*) and ARG (encoded by *ABL2*), both of which link extracellular stimuli to signaling pathways to control cell survival, growth, and migration [26]. Although the two genes are highly conserved in the N-terminal kinase domain and the C-terminal, c-Abl and ARG have distinct cellular localizations and specialized functions [26]. c-Abl mediates the DNA damage-repair response through its nuclear localization signals and a DNA binding domain, whereas ARG has cytoskeletal remodeling functions by its additional binding capacity for actin and microtubules [27]. Despite the oncogenic role that Bcr-Abl plays in hematopoietic malignancies like CML, activated c-Abl has been reported to be involved in the progression of solid tumors such as non-small cell lung cancer (NSCLC) [39] and breast cancer [30, 31]. However, nuclear c-Abl has also been reported to be involved in DNA damage response and to contribute to apoptosis [40, 41]. Due to the contradictory experimental evidence regarding the cellular function of c-Abl, the role that the c-Abl tyrosine kinase plays in cell proliferation and cell survival is controversial [42]. As shown in Figure 3B, high expression of c-Abl correlates with poor outcome in NB patients. Moreover, we found that the full-length c-Abl but not the Bcr-Abl fusion protein was detected in IMR-32, NB-19, SH-SY5Y and SK-N-AS cell lines (Figure 3C). In addition, bosutinib inhibited the phosphorylation of c-Abl both *in vitro* and *in vivo*, suggesting that c-Abl inhibition may also play a role in the anti-tumor effect of bosutinib in NB. However, given the fact that there are conflicting evidence about the biologic functions of c-Abl in solid tumors including NB, further study is needed to explore how the inhibition of c-Abl contributes to bosutinib-induced toxicity in NB.

Chemoresistance in cancer patients is the main clinical obstacle that is responsible for relapse. In this study, we examined whether bosutinib could sensitize NB cells to the treatment of traditional therapeutic agents: Dox and VP-16. We found that bosutinib significantly enhanced Dox- and VP-16-induced cytotoxic effects on IMR-32 and SH-SY5Y cells (Figure 4A). Consistently, bosutinib augmented cell apoptosis induced by Dox- and VP-16 in IMR-32 and SH-SY5Y cells (Figure 4B). Our results suggest that the combination of bosutinib and Dox or VP-16 may achieve better outcomes than single drug treatment in NB therapy.

Imatinib (Gleevec, STI571), the first-generation tyrosine kinase inhibitor of Bcr-Abl, has been approved by FDA for the treatment of Ph+ CML and gastrointestinal stromal tumors (GIST) [43, 44]. Despite the success of imatinib in treating CML, an alternate therapy is needed in approximately one third of CML patients [45]. The demand to treat patients with imatinib-resistant Bcr–Abl mutants led to the development of second-generation tyrosine kinase inhibitors such as nilotinib (Tasigna, AMN107), dasatinib (Sprycel, BMS-54825) and bosutinib (Bosulif, SKI-606). Dasatinib, nilotinib and bosutinib potently inhibit most of imatinib-resistant mutations except the T315I gatekeeper mutant [46], whereas the third-generation tyrosine kinase inhibitor SGX393 and ponatinib (Iclusig, AP24534) show strong inhibition in imatinib-resistant T315I mutant [46]. Compared with imatinib, which recognizes the inactive conformation of c-Abl, bosutinib targets the active conformation of c-Abl's the kinase domain [46]. Bosutinib inhibits the kinase activity of both c-Abl and Bcr–Abl fusion protein at a much lower IC_50_ than imatinib. Besides, bosutinib also inhibits Src kinase activity, which contributes to its anti-tumor efficacy. Notably, treatment with imatinib did not affect the phosphorylation of c-Abl on Tyr-245 in NB cells [33], whereas bosutinib significantly abrogated p-c-Abl (Y245) level in a subset of NB cells used in this study and in SH-SY5Y xenografted NB tumor tissue. Therefore, our data shed light on further study regarding the role of c-Abl inhibition in bosutinib-induced toxicity in NB.

Collectively, our data indicate that the small molecule bosutinib exerts potent anti-tumor efficacy in NB by suppressing the activities of Src, c-Abl and the downstream signaling pathways both *in vitro* and *in vivo*. Taken together, our study suggests that Src and c-Abl are potential therapeutic targets in NB and that novel Src/Abl inhibitors like bosutinib alone or in combination with other chemotherapeutic agents may benefit NB patients.

## MATERIALS AND METHODS

### Antibodies and reagents

The dual Src/Abl inhibitor, bosutinib, was purchased from LC Laboratory (B-1788, Woburn, MA, USA). Doxorubicin (dox, D1515), etoposide (VP-16, E1383), and anti-β-Actin antibody (A2228) were purchased from Sigma (Sigma-Aldrich Corp, St. Louis, MO, USA). The antibodies against p-Src (Y416, 6943S), Src (2108S), p-c-Abl (Y245, 2868S), c-Abl (sc-56887), p-STAT3 (Y705, 4904S), STAT3 (4904S), p-ERK (T202/Y204, 9106L), ERK (4695S), p-S6 (Ser235/236) ribosomal protein (4858S), S6 ribosomal protein (2217S), Caspase 3 (9662S), PARP (9532S), and anti-Mouse (7076S) or anti-Rabbit (7074S) IgG were purchased from Cell Signaling Technology (Danvers, MA, USA).

### Cell lines and cell culture

Six human NB cell lines were used in this study, including three MYCN-amplified (IMR-32, NGP, NB-19) and three MYCN-non-amplified (CHLA-255, SH-SY5Y, SK-N-AS) cell lines. These cells were all cultured in RPMI Medium 1640 (Lonza, Walkersville, MD, USA) supplemented with 10% (v/v) heat-inactivated Fetal Bovine Serum (FBS, SAFC Biosciences, Lenexa, KS, USA), 100 units/mL penicillin, and 100 μg/mL streptomycin. Cells were cultured at 37°C in a humidified atmosphere of 5% CO_2_. The SH-SY5Y cell line with stable expression of luciferase was generated by transfection with a pcDNA3-luciferase expression plasmid into the cells. A stable cell line was established after 10 days of 800 μg/ml G418 selection (Enzo Life Sciences, Farmingdale, NY, USA).

### Cell viability assay

Cell Counting Kit-8 (CCK-8, WST-8[2-(2-methoxy-4-nitrophenyl)-3-(4-nitrophenyl)-5-(2,4-disulfophenyl)-2 H-tetrazolium, monosodium salt]) (Dojindo Laboratories, Rockville, MA, USA) was used to conduct the assays. The cell viability assay was performed as previously described [47, 48]. Briefly, cells were seeded in 96-well plates at a density of 1 × 10^4^ cells per well. After 24 hrs of incubation at 37°C, cells were either allowed to grow in medium alone or in increasing concentration of bosutinib, Dox, VP-16, or a combination of bosutinib with Dox or VP-16 and were incubated for 24 or 48 hrs, depending on the experiment. Then a mixture of 10 μL of CCK-8 and 190 μL of RPMI with 10% FBS was added to each well and incubated for one hour. Then, the microplate reader was used to measure the absorbance of each well at 450 nm.

### Cell imaging

All the NB cell lines tested were seeded in 96-well plates at a concentration of 1 × 10^4^ cells per well. After 24 hrs or 48 hrs of treatment with bosutinib using the indicated concentrations, an optical microscope was used to capture the cell morphologies. Each experiment was repeated six times.

### Colony formation assay

Cell anchorage-independent growth capabilities were evaluated by soft agar assays, which were performed as described previously [49, 50]. Briefly, in 6-well plates, the bottom layer was made by mixing 2 mL of 0.5% agar/RPMI solution in each well and allowing the solution to cool to a semi-solid. For the upper layer in each well, cells were mixed with 1.5 mL of 0.3% agar at 1 × 10^4^ cells per well with the indicated concentrations of bosutinib. Cells were allowed to grow at 37°C for two to three weeks until the colonies were visible to the naked eye. Cells were then stained with crystal violet dye for four hours (C3886, Sigma). Then images were captured with the optical microscope and colonies were counted. Each assay was performed in duplicate.

### Protein immunoblotting

For the protein immunoblotting (Western blot) assays, the experiments were performed as described previously [51–53]. After the indicated treatments, cells were washed twice with ice cold PBS. The cell pellets were then dissolved in RIPA lysis buffer (50 mM Tris-HCl at pH 7.4, 150 mM NaCl, 1 mM EDTA, 1% NP-40, 0.25% sodium deoxycholate) with protease inhibitors (1 mM phenylmethylsulfonyl fluoride, 1 mM benzamidine, 10 μg/mL leupeptin, 1 mM dithiothreitol, 50 mM sodium fluoride, 0.1 mM sodium orthovanadate) and phosphatase inhibitors (phosphatase inhibitor cocktail 2 and 3 (P5726 and P0044, Sigma)) for 30 min at 4°C. The solutions were then centrifuged at 13000 RPM for 15 min, and the supernatants were collected. Measurements of protein concentrations were performed using a Bradford reagent (Bio-Rad Laboratories, Hercules, CA, USA). 4X loading buffer mixed with each sample, and samples were heated at 100°C for 7 min. The cell lysates were then subjected to 10% or 15% SDS-PAGE electrophoresis and transferred to polyvinylidence fluoride (PVDF) membranes and blocked with 5% milk at RT (25°C) for one hour. The membranes were then incubated with primary antibodies at 4°C overnight. The following day, the membranes were incubated with horseradish peroxidase-conjugated secondary antibodies against rabbit or mouse IgG at room temperature for one hour. The membranes were developed using the ECL-Plus Western blotting system (GE Health Care, Buckinghamshire, UK). β-Actin was used as a loading control for whole cell extracts in all experiments.

### Orthotopic mouse model of NB

In this study, NCR nude mice (Taconic, Hudson, NY, USA) were used. These mice were kept under obstacle conditions (pathogen-free environments offered by plastic cages with sealed air filters). Orthotopic internal implantation of NB cells was conducted to establish the NB mouse model as previously described [54–56]. Briefly, a solution of 0.1 ml of PBS containing 1.0 × 10^6^ human luciferase-transduced SH-SY5Y cells was prepared. Then, a transverse incision over the mouse's left flank was made, and these cells were surgically injected into the left renal capsule at the superior pole of the left kidney.

After allowing the injected cells to engraft for two to three weeks, a bioluminescent imaging system was employed to monitor tumor growth. Tumor-bearing mice with similar tumor size were randomly divided into two groups, a DMSO control group and a bosutinib treated group (30 mg/kg by intraperitoneal (i.p.) injection once daily for 21 days).). Each group contained three mice. All mice were sacrificed at the end of the treatment, and the tumors and right kidneys were harvested, weighed and photographed.

For the protein immunoblotting of the tumor tissues, another set of SH-SY5Y-luciferase xenografted mice with similar sizes were treated with either DMSO or bosutinib (30 mg/kg by i.p. injection) daily for two days. The mice were sacrificed at the end of treatment and the tumors were collected and lysed for immunoblotting. All mice were handled according to protocols approved by the Institutional Animal Care and Use Committee of the Baylor College of Medicine.

### Statistical analysis

All values were presented as mean ± standard deviation (SD). A two-tailed Student's t-test was used to determine the statistical significance of *in vitro* and *in vivo* assays between the control and drug treatment groups. Each assay was repeated at least twice, and representative results were presented. *P*<0.05 was considered to be statistically significant.
